# Deciphering the Counterplay of *Aspergillus fumigatus* Infection and Host Inflammation by Evolutionary Games on Graphs

**DOI:** 10.1038/srep27807

**Published:** 2016-06-13

**Authors:** Johannes Pollmächer, Sandra Timme, Stefan Schuster, Axel A. Brakhage, Peter F. Zipfel, Marc Thilo Figge

**Affiliations:** 1 Research Group Applied Systems Biology, Leibniz Institute for Natural Product Research and Infection Biology – Hans Knöll Institute, Jena, Germany; 2 Faculty of Biology and Pharmacy, Friedrich Schiller University Jena, Germany; 3 Department of Bioinformatics, Faculty of Biology and Pharmacy, Friedrich Schiller University Jena, Germany; 4 Department of Molecular and Applied Microbiology, Leibniz Institute for Natural Product Research and Infection Biology – Hans Knöll Institute, Jena, Germany; 5 Department of Infection Biology, Leibniz Institute for Natural Product Research and Infection Biology – Hans Knöll Institute, Jena, Germany

## Abstract

Microbial invaders are ubiquitously present and pose the constant risk of infections that are opposed by various defence mechanisms of the human immune system. A tight regulation of the immune response ensures clearance of microbial invaders and concomitantly limits host damage that is crucial for host viability. To investigate the counterplay of infection and inflammation, we simulated the invasion of the human-pathogenic fungus *Aspergillus fumigatus* in lung alveoli by *evolutionary games on graphs*. The layered structure of the innate immune system is represented by a sequence of games in the virtual model. We show that the inflammatory cascade of the immune response is essential for microbial clearance and that the inflammation level correlates with the infection-dose. At low infection-doses, corresponding to daily inhalation of conidia, the resident alveolar macrophages may be sufficient to clear infections, however, at higher infection-doses their primary task shifts towards recruitment of neutrophils to infection sites.

The great efficiency of the human immune system with regard to the recognition and elimination of infectious microbes is due to its layered and redundant structure and its well-orchestrated response across elevating levels. For example, the complex immune system is divided into the two main interacting levels of innate and adaptive immunity. Many infectious microbes are cleared already by the innate immune response that is immediately active but only grossly specific^1^. However, if the infectious microbe cannot be cleared in this way, inflammatory signals elevate the response from the level of innate immunity to the level of adaptive immunity. Activation of an adaptive response, *e.g.* involving affinity maturation of antibodies by B-cells in germinal centres, takes days to weeks, however, at the benefit of being highly specific for the invader^2^.

The layered structure of the immune system is also recognised within the levels of innate and adaptive immunity itself. For example, adaptive immunity does not necessarily require to mount the tedious process of antibody affinity maturation, if the infectious microbe was encountered before and can be defeated by the activation of previously generated memory cells that directly go into production of the highly specific antibodies^2^. Similarly, innate immunity comprises the immediate response of humoral immunity, like the complement system, that may eliminate the infectious microbes by opsonisation associated with inflammatory signalling to recruit and activate phagocytic cells within a few hours and/or the formation of membrane attack complexes^3^. Furthermore, one can distinguish phagocytic cells that are resident in organs from those that are recruited in support from the bloodstream to the site of infection within tens of hours^1^.

Apart from the fact that the layered structure of the immune system is beneficial for the strength and efficiency of the response, it is obvious that an elevating regulation between infection and inflammation is beneficial in being protective for the host itself. The danger for the human host due to dysregulation of the immune response becomes impressively evident in the case of sepsis, *i.e.* a whole-body inflammatory response to an infection that may induce multi organ failure^4^. Thus, as important as it is that the human host responds with inflammation against infection, it is mandatory for the protection of the host against its own immune system to avoid unnecessary overshoots and to ensure a proper shut down of the response after infection clearance^5^. In what follows, we refer to the pathogen-induced infection and the host-driven inflammation, which are opposing actions from positions of defence, as *counterplay*.

Virtual infection-inflammation models can be constructed by different modelling techniques like for example by *ordinary differential equation models* (ODE), *state-based models* (SBM), *agent-based models* (ABM) and game theory. The purpose of a theoretical study and the complexity of a biological system determine how suitable a certain approach is.

ODE allow for straightforward modelling and have previously been applied in the context of bacterial infections^6^,^7^,^8^,^9^ as well as for *A. fumigatus* infection^10^. However, ODE assume spatially homogeneous environments, where the constituents occur in high concentrations, making them inappropriate for a realistic simulation of the infection-inflammation scenarios of *A. fumigatus* conidia in the lung. SBM and ABM allow for the stochastic simulation of biological systems at the level of single events^11^,^12^. While SBM neglect spatial resolution, ABM simulate single individuals within a real spatial structure that may be represented on a grid or in continuous space. For example, a virtual infection-inflammation model of the human-pathogenic fungus *Candida albicans* in human blood was recently formulated in terms of a bottom-up modelling approach using SBM and ABM for quantitative parameter estimation^13^,^14^. Another ABM was developed for investigation of spatio-temporal dynamics of *A. fumigatus* infection of *in vitro* experiments^15^ and in a virtual human alveolus with regard to inflammation-induced chemotaxis of AM^16^. The latter model was extended to a hybrid ABM that enabled investigation of molecular diffusion using *partial differential equations* to decipher quantitative properties of candidate chemokines^17^.

The aforementioned modelling techniques simulate the time course of an underlying biological system by means of mechanistic processes. As a consequence, the dimensions of the parameter space are rapidly increasing and, since many of these parameters are unknown to date, a rigorous parameter estimation may render these approaches infeasible^14^. Therefore, to investigate the innate immune response against *A. fumigatus* in the human lung at the humoral and cellular level, we here pursue a game-theoretical approach that resolves the counterplay of infection and inflammation at the different levels.

*Game theory* is a mathematical concept of optimisation and the main advantage of this approach is that different effector mechanisms can be elegantly condensed into a reduced number of combined parameters. This renders computational simulations tractable and still allows for the identification of the key parameters that deserve particular attention in future experimental investigations. *Classical game theory* (CGT) was originally developed as decision support^18^ in interactive situation in which the aims, goals and preferences of individual actors, *i.e.* the players of a game, are in conflict with each other^19^. The decisions available to the players correspond to strategies from which they can choose. Based on its own strategy and the strategies of the other players with which interactions are carried out players receive a benefit – also termed *payoff*^20^. However, each player in CGT was assumed to act perfectly rationally, to have the complete set of information about all possible outcomes of the game and their related payoffs as well as the cognitive capabilities to deduce from this information the optimal strategy^19^. To represent phenomena observed in interactions between living organisms, the paradigm of CGT turned out to be too restrictive.

The restrictions were softened by the concept of *evolutionary game theory* (EGT)^21^. This concept allowed for more flexibility and – inspired by evolution theory – implemented irrational behaviour, *e.g.* in terms of mutation. Thus, players do not simply optimise their payoff with regard to their strategic alternatives; rather, they replicate, survive or die based on the fitness associated with their respective strategy^22^. As a consequence, the dynamics of strategies involves a series of iterative evolutionary steps, a feature completely missing in CGT^19^. Moreover, the field of *evolutionary games on graphs* emerged, where not all players interact with each other, but the number of interactions is reduced to the ones that can be reasoned for by neighbourship relations between individuals, *e.g.* based on spatial proximity, social connections or genetic relationships^19^. There have been several contributions to the field of fungal infections using game-theoretic approaches in the recent past^23^: Hummert *et al*. investigated the cooperative behaviour between *Candida albicans* cells that were phagocytosed by macrophages^24^. In another study on *C. albicans* infection, the dynamics of the yeast-to-hyphal transition in response to various actions of the host was investigated by EGT^25^. It was found that the host requires a differentiated response to the distinct morphological states of *C. albicans* in order to keep the fungus in the least pathogenic state. Other examples, going beyond the context of fungal infections, have been reviewed by Hummert *et al*. regarding cellular interactions^20^ and by Bohl *et al*. regarding molecular interactions^26^.

In this study, we theoretically investigate the counterplay of infection and inflammation during the innate immune response against invading microbes by applying EGT. The main advantages of this stochastic modelling approach are that (i) systems with a small number of constituents are adequately captured, (ii) model parameters are reduced by a relative representation of processes, and (iii) spatial system resolution can be realized on a graph. While such a modelling approach is generally applicable to infection-inflammation scenarios caused by any kind of infectious microbe, for illustrative purposes we here focus on the concrete example of the saprophytic human-pathogenic fungus *Aspergillus fumigatus*^27^. This fungus represents a prime example, because humans inhale several thousands of its ubiquitously present airborne conidia every day^28^. Thus, while this fungus constitutes a common challenge to the human host, its potential threat can obviously be permanently managed under physiological immune conditions. This implies that an innate immune response against this infectious agent is generally sufficient, involving moderate inflammatory conditions that are usually mounted unnoticed in immunocompetent individuals. However, the situation is different for immunocompromised individuals and/or when high fungal doses are inhaled, in which case the pathogen can become a major cause of life-threatening infections^29^. The immune system of immunocompromised individuals may not be able to clear the lung from *A. fumigatus* conidia in due time, before the onset of conidial germination that may be followed by invasive growth^30^. Inhaled conidia typically are in a resting state, covered by a rodlet layer that renders them immunologically inert^31^. However, taking advantage of nutrient-rich conditions in the lung alveoli, resting conidia may swell and germinate, ultimately leading to the formation of hyphae and the ability of the fungus to penetrate tissue and eventually enter the bloodstream, disseminating through the whole body. As a consequence, invasive pulmonary aspergillosis is associated with high mortality rates ranging from 30–90%^32^.

On invading the human organism, fungal cells are partly intercepted by mucosal epithelial cells, however, due to their small size of about 2–3 μm, significant amounts of the conidia can enter the lower respiratory tract of the lung (see Fig. 1(a)). They end up in so-called *alveolar sacs* (AS)^33^ that consist of alveoli, which are the smallest units of the human lung^27^. Alveoli exist in various polyhedral shapes with a surface to volume ratio that optimises the gas exchange through their thin epithelial layers^34^. Once conidia enter the alveoli, they are embedded within the nutrient-rich and highly viscous alveolar lining layer, also called pulmonary surfactant, where they are faced with several defence mechanisms of the host^27^,^35^. During the innate immune response different sequential phases can be involved. Depending on how successful the pathogen can be cleared from the human host in one phase, the next phase, which is associated with a higher level of inflammation, may be initiated. As depicted in Fig. 1(b), the innate immune response against *A. fumigatus* conidia comprises humoral factors, like the complement system that is subject of Game I, and phagocytic cells, such as *alveolar macrophages* (AM) and *polymorphonuclear neutrophils* (PMN) being subjects of Game II and III, respectively.

As soon as conidia get into contact with the surfactant, complement is activated and proteins start to opsonise their surfaces, with amounts of bound complement proteins being proportional to the surface area of the fungal cell^36^. Opsonisation by complement proteins induces inflammation at the site of infection and the release of chemoattractants, *e.g.* C3a and C5a^3^. In addition to that, opsonisation of the fungal surface increases phagocytosis and the production of reactive oxygen intermediates (ROI) by AM and PMN enhancing phagocyte activation on direct physical contact with the pathogen^30^. Taken together, while the complement system drives the innate immune response against *A. fumigatus* by inflammatory signals, the formation of terminal membrane attack complexes are expected to be prohibited by the thickness of the cell wall^37^.

Upon contact of *A. fumigatus* conidia with type II *alveolar epithelial cells* (AEC), these cells secrete inflammatory molecules, such as IL-6 and TNF-*α* as well as the chemoattractant IL-8^38^. AM are resident phagocytes in lung alveoli and are the first immune cells that get into contact with inhaled pathogens like *A. fumigatus* conidia^34^,^39^. They are able to phagocytose both resting and swollen conidia, but only swollen conidia are effectively killed intracellularly^39^. AM lack the ability to phagocytose the hyphal morphotype of *A. fumigatus*, but are critical for hyphal recognition because this leads to the chemotactic recruitment of circulating phagocytes, like PMN, from the bloodstream to the site of infection^29^,^40^.

PMN phagocytose and kill conidia but also hyphal structures. If hyphal structures are too large for being phagocytosed, PMN may release the content of their granules into extracellular space^30^. These extracellular factors are involved in killing hyphae, but also cause damage to the surrounding host tissue^27^. Furthermore, PMN may commit an altruistic suicide as a final act of defence against hyphae, thereby releasing DNA fibers into the environment that may trap the fungus^41^. These so-called *neutrophilic extracellular traps* (NETs) were shown to have fungistatic rather than fungicidal effects^41^. AM and PMN are thought to be key players in the defence against *A. fumigatus* and an impairment of either phagocyte type is commonly associated with a relatively high susceptibility for severe infections by this fungus^42^.

As is clear from the example of the human-pathogenic fungus *A. fumigatus*, investigating the counterplay of infection and inflammation is highly important in order to gain insights into the complex host-pathogen interactions that may ultimately reveal possibilities for therapeutic interventions. However, since it is virtually impossible to investigate these processes in alveoli of the human lung *in vivo* and under physiological conditions^16^, we here pursue a systems biology approach starting with theoretical modelling. We apply EGT on graphs to investigate the counterplay of pathogen-induced infection and increasing levels of host inflammation during *A. fumigatus* infection in AS of the human lung according to the time course depicted in Fig. 1(b). The model is constructed on the firm basis of experimental data available today (see Methods section) and is applied to generate new hypothesis that can direct future experimental investigations (see Discussion section).

The present study, using infections by the human-pathogenic fungus *A. fumigatus* as an example, reveals the importance of the immune response to be tightly regulated and that this regulation is realised by the layered structure of the immune system. In particular, our simulations reconcile the contradictory view on the role of AM in the immune response^43^,^44^: We observe that AM have a key role in the regulation of the first steps of the immune response and that this regulation depends on the strength of the infection-dose. While the phagocytic activity of AM is sufficient to limit low infection-doses – corresponding to values of daily inhalation – inflammatory signalling by AM for recruitment of PMN becomes their primary task at higher infection-doses.

## Results

**Figure 1:**
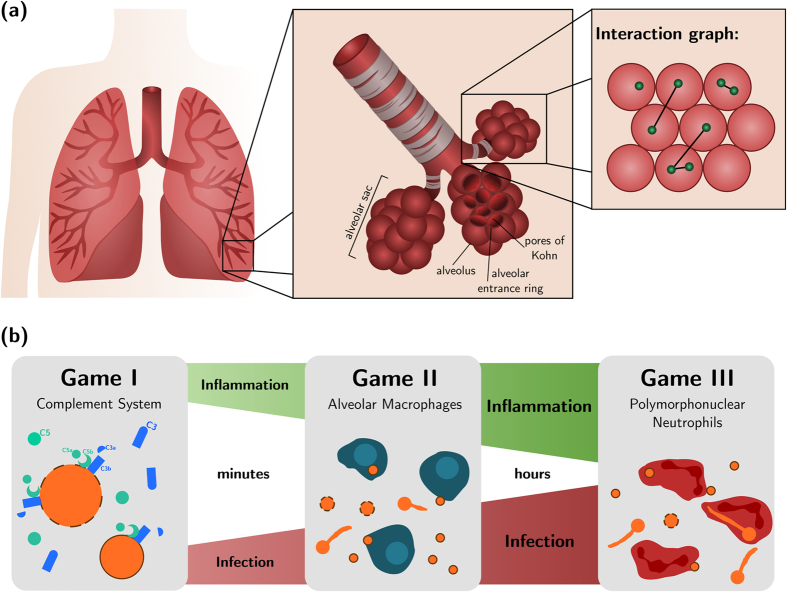
Lung architecture and design of evolutionary games. (**a**) Organisation of densely packed alveoli in alveolar sacs in the lower respiratory tract of the lung. An example distribution of eight fungal cells (shown in *green*) over a cross-sectioned alveolar sac and their proximity-based interactions (*black* connections). (**b**) Model design of the consecutive sequence of evolutionary games on graphs for the innate immune response against *A. fumigatus* infection in the lung.

In this section, we first analyse the degree of proximity between conidia of *A. fumigatus* in AS of the human lung. Based on the derived interaction graphs between fungal cells, we then consider the results of three evolutionary games representing distinct stages of the innate immune response against this human-pathogenic fungus (see Fig. 1(b)). These stages comprise the humoral response by the complement system (Game I), the cellular immune response by AM (Game II) and PMN (Game III). Finally, by linking these three individual games in a time-ordered fashion, we present the results of infection-inflammation scenarios in the human host. To quantify the infection-inflammation scenarios, we defined the infection score (see Eq. 6) and normalised inflammations that mediate between Game I and II (see Eq. (7)) and Game II and III (see Eq. (8)).

### Virtual infection-inflammation model reveals degree of fungal interactions in alveolar sacs

**Figure 2:**
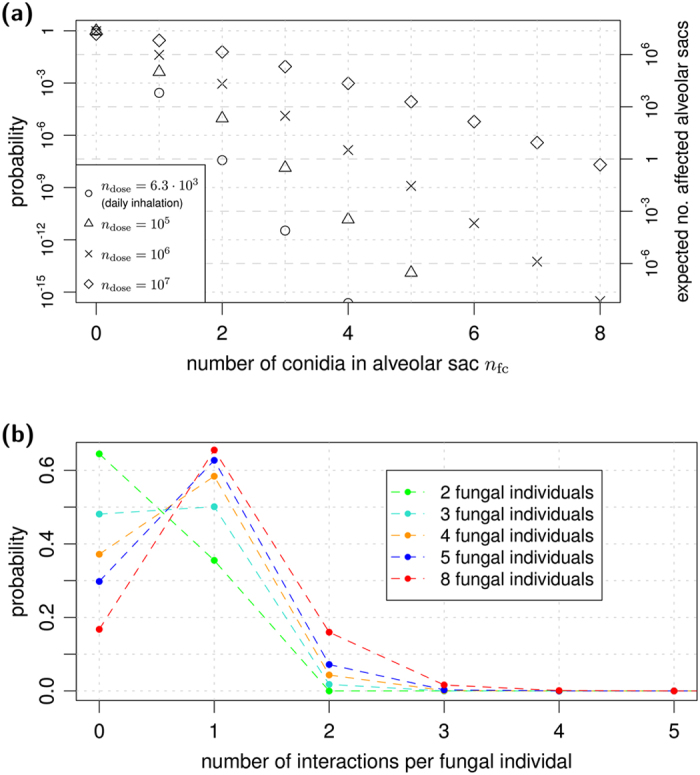
Number of conidia and their interactions per alveolar sac. (**a**) Dose-dependent probability distribution for the number of conidia entering the alveolar sac. Rescaling of the probability values were used to determine the expected number of affected alveolar sacs in the human lung, as shown by the scale on the right hand side. (**b**) Probability distribution for the number of interactions per fungal individual depending on the number of conidia inserted into the alveolar sac. Distributions were obtained from 10^6^ interaction graphs.

We performed *in silico* experiments to simulate the counterplay of *A. fumigatus* infection and host inflammatory response by evolutionary games on graphs. A graph defines the proximity-based interactions between fungal cells in AS of the human lung (see Fig. 1(a)). Each game of the infection-inflammation scenario represented a consecutive sequence of three stages of innate immunity (see Fig. 1(b)). Within each AS, consisting on average of 21 densely packed alveoli, the drawn number of conidia *n*_fc_ were uniformly distributed over its alveoli. As shown in Fig. 2(a), we found that irrespective of the fungal dose, most likely there will be no conidium at all in a randomly chosen AS. However, of all the AS that contained fungal cells, their number clearly depended on the fungal dose and was associated with quite different upper limits of conidia that could be expected in the AS. For a fungal dose that corresponds to daily inhalation (6.3 · 10^3^ conidia), two conidia per AS are conceivable, whereas for extremely high doses (10^7^ conidia) up to eight conidia may be encountered in a single AS.

Fungal individuals in the same AS may become engaged in a game among each other under the influence of the immune response at a certain stage. As described in the Methods section, the graph-based approach defines interactions between fungal cells, where the number of interactions per fungal cell depends on their number in the corresponding AS. As shown in Fig. 2(b), this analysis revealed that two fungal cells in an AS most likely stay solitary, whereas three to eight individuals in an AS typically lead to one expected interaction per conidium.

### Complement activity drives emergence of fungal morphotypes

**Figure 3:**
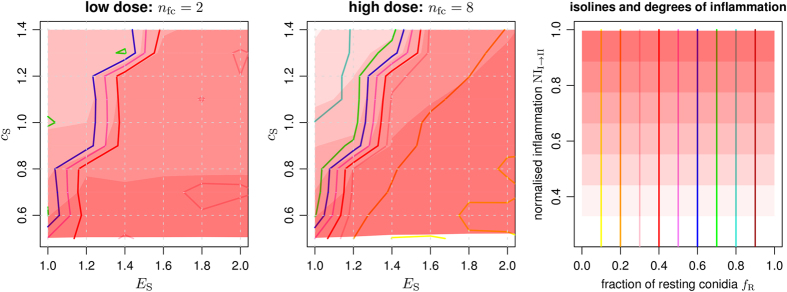
Fractions of *A. fumigatus* morphotypes and degrees of inflammation in response to the complement system. Equilibrium fractions of conidia in the resting state were determined from averaging over 100 repetitions per parameter configuration. Parameters were scanned in a lattice-based fashion at distinct points of the parameter space. Isolines (*colored lines*) for fractions of conidia in the resting state and isobands (*red shaded areas*) for different degrees of inflammation were generated using a modified version of the marching-squares algorithm for bilinear interpolation over rectangular grids. Parameters *E*_R_ = 1, *c*_R_ = 0.5 were kept fixed.

In Game I, different morphotypes emerge from the interaction of fungal cells with the complement system that are shown in Fig. 3 for different parameter combinations and for low (*n*_fc_ = 2) and high (*n*_fc_ = 8) infection-doses. As could be expected, with rising nutrient availability *E*_S_ for swollen conidia or diminishing complement effects *c*_S_ against swollen conidia, their fraction increases over that of resting conidia, *f*_S_ > *f*_R_. Regions with comparable fractions of resting and swollen conidia, *f*_R_ ≈ *f*_S_, were found for both infection-doses, however, these regions were more extended for the lower infection-dose. At high infection-doses, where conidia had on average more interaction partners per alveolus, it was frequently observed that one of the two populations dominated over the other one with a fraction above 80 %. This effect could be attributed to the dose-dependent impact of adaptation and mutation events in each simulation time-step. For low relative to high infection-doses, mutations occur more often than adaptation events due to a lack of interaction neighbours among the widely separated fungal cells.

### Alveolar macrophages reach phagocytic limits for high fungal doses

**Figure 4:**
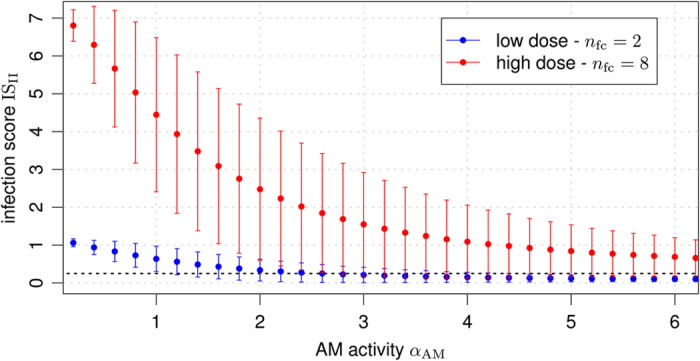
Dose-dependent infection scores according to simulations of Game II. The average infection score IS_II_ over all parameter combinations shown as a function of AM activity. Error bars denote one standard deviation. The *black short-dashed line* marks the infection score threshold IS = 0.25 below which clearance of infection is achieved.

We performed simulations of Game II to identify the most relevant parameters under the impact of the immune response by AM. Using the concept of mutual information, as explained in the Methods section, we found that the activity of AM was of central importance regarding the infection score IS_II_ (see Eq. (6) and Table S2). Our investigations revealed that AM were in the position to keep the fungal cells in check for low infection-doses of fungal cells and for sufficiently high phagocytosis activity of AM. However, as shown in Fig. 4, AM were not able to remove the infectious agents in high-dose scenarios due to their inability to counteract hyphal cells appropriately. It can be expected that their function regarding elevation of the immune response via inflammatory and chemoattracting signals plays an important role in this case.

### Recruitment of neutrophils can be essential for successful clearance

**Figure 5:**
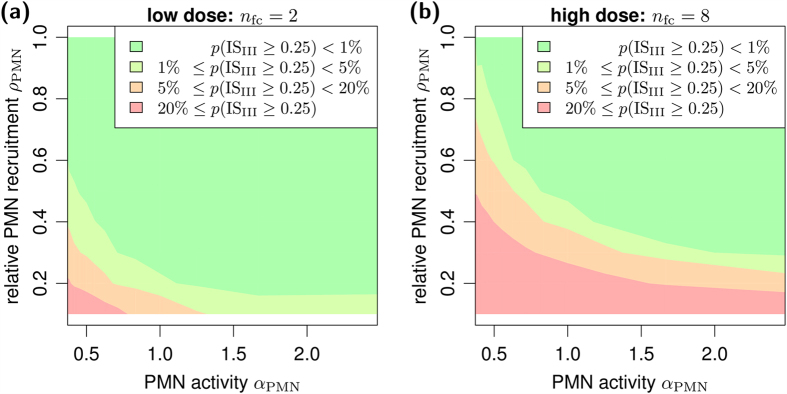
Dose-dependent connection between infection score and PMN parameters. Landscape of the persistent infection probability for different fungal doses in scenarios with (**a**) low and (**b**) high infection-doses. Isobands (*colored areas*) for different probability ranges were generated using a modified version of the marching-squares algorithm for bilinear interpolation over rectangular grids.

In Game III, *A. fumigatus* conidia were faced with the professional phagocytes PMN. First, we identified the most relevant parameters by computing the mutual information between each of the game’s parameters and the infection score (see Eq. 6). The PMN recruitment *ρ*_PMN_ and phagocytosis activity *α*_PMN_ against live fungal cells were found to be most relevant for the outcome of the infection (see Table S2). We further analysed these parameters for low and high infection-doses and the results are shown in Fig. 5. It should be noted that the infection score IS_III_ = 0.25 represented the special case of one resting conidium in the AS that survived the immune response of the host. Consequently, infection scores IS_III_ < 0.25 and IS_III_ ≥ 0.25 represent, respectively, clearance and persistence of the *A. fumigatus* infection. The measure *p*(IS_III_ ≥ 0.25), plotted in Fig. 5, denotes the fraction of parameter combinations over all performed simulations for which the fungal infection persisted in the host. We identified the most important parameters for infection clearance and found that, for a low number of infectious agents, PMN were able to clear the infection in most of the cases, whereas reduced recruitment and activity of PMN eventually led to the persistence of infection. In the limit of high infection-dose, PMN activity and a sufficiently high recruitment were pivotal to remove *A. fumigatus*. We further found that a reduced PMN activity could be partly compensated by an increase in the PMN recruitment. Moreover, independent of the precise value of PMN activity, sufficient PMN recruitment was generally found to be an essential prerequisite for successful infection clearance.

### Infection-inflammation scenarios reveal regulatory role of alveolar macrophages

Going beyond the separated analysis of evolutionary games for the three distinct stages of the innate immune response against the human-pathogenic fungus *A. fumigatus*, we linked the games in a time-ordered fashion to simulate infection-inflammation scenarios in the human host. Thus, starting with Game I, we determined the infection score IS_I_ (see Eq. 6) due to the immune response by the complement system. Next, we linked Game I to Game II by computing the normalised inflammation NI_I→II_ (see Eq. (7)), which determined AM activity in the second stage of the immune response. The infection-inflammation scenario at this stage was evaluated by the infection score IS_II_ (see Eq. 6) and linking to Game III was realised by the normalised inflammation NI_II→III_ (see Eq. (8)), which determined PMN recruitment in the third stage of the immune response and gave rise to the infection score IS_III_ (see Eq. 6 and Fig. 1(b)).

**Figure 6:**
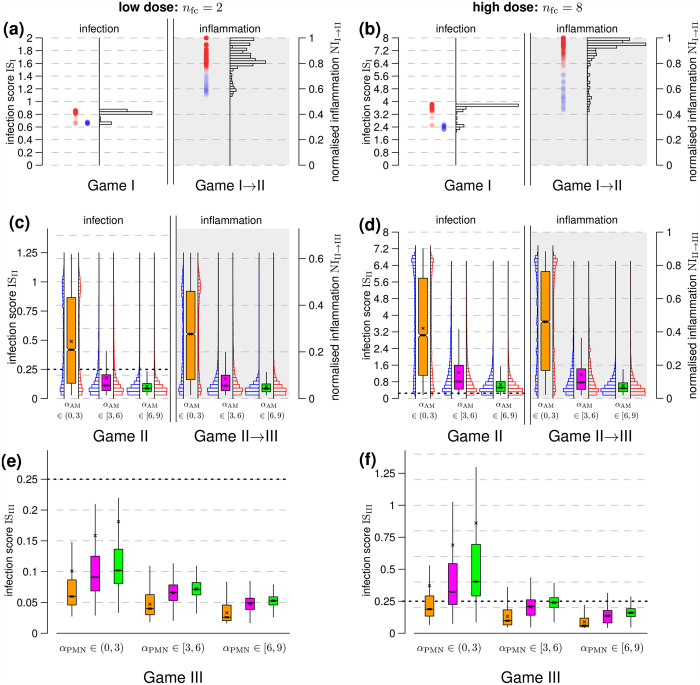
Dose-dependent relation between infection and inflammation across the consecutive sequence of evolutionary games. Two infection-inflammation scenarios were simulated: low infection-dose in (**a**,**c**,**e**) and high infection-dose in (**b**,**d**,**f**). In (**a**,**b**) the distribution of infection scores and corresponding values for the normalised inflammation are shown as *black histograms*. The distribution of higher (lower) values for the normalised inflammation with NI_I→II_ ≥ 0.75 (NI_I→II_ < 0.75) are shown by circles colored in *red* (*blue*). The inflammatory signal NI_I→II_ was fed into Game II scaling AM activity. The effects of high and low values for the normalised inflammation are shown in (**c**,**d**) using *red* and *blue* inline histograms grouped by different levels of AM activity. The *red* and *blue* inline histograms were combined in the *notched box plots* and shown in *orange*, *violet* and *green* color, respectively, for low, medium and high AM activity with average values marked by (×). (**e**,**f**) Connection between Game II and Game III was set by the recruitment parameter *ρ*_PMN_ = NI_II→III_. Distributions for the different AM activities are presented in the same colors as in (**d**,**e**) grouped by PMN activities. The *black short-dashed lines* in (**c**–**f**) mark the infection score threshold IS = 0.25 below which clearance of infection is achieved.

The simulation results for low and high infection-doses as well as for varying AM and PMN activities are presented in Fig. 6. The counterplay between *A. fumigatus* infection and the complement system (Game I) yielded values for the infection score and the normalised inflammation that are shown in Fig. 6 for (a) low (*n*_fc_ = 2) and (b) high (*n*_fc_ = 8) infection-doses. The infection score IS_I_ resulting from Game I is depicted on the left-hand side in the black histograms in Fig. 6(a,b), while the corresponding normalised inflammation NI_I→II_ is shown on the right-hand side. The normalised inflammation revealed a bimodal distribution that subdivides all simulations into infection-inflammation scenarios with low (blue) and high (red) NI-values. Interestingly, a comparison with the corresponding classes for the infection score did not show this clear separation. This result was observed for both low and high infection-doses and an in-depth analysis revealed that, while low (blue) inflammation values were associated with lower infection scores, higher (red) inflammation values were not only associated with higher infection scores but, quite unexpectedly, also with lower infection scores. It turned out that the reason for this behavior is in the effect that the complement system exerted on resting and swollen conidia. As can be observed in Fig. 3, high inflammation (dark red areas) exist for *f*_R_ > 0.5 and corresponds indeed to lower infection scores where the fraction of resting conidia is larger than that of swollen conidia. Furthermore, it could be observed that these cases were associated with approximately equal complement activity against resting and swollen conidia (*c*_R_ ≈ *c*_S_) in combination with only small differences between the nutrient contributions *E*_R_ and *E*_S_.

The normalised inflammation NI_I→II_ that was induced by the response of the complement system (Game I) had direct impact on AM activity in Game II. The corresponding results are depicted in Fig. 6 for (c) low and (d) high infection-doses. In both cases, the highest impact on the infection scores could be attributed to the AM activity *α*_AM_, where higher AM activity was associated with more strongly reduced infection and vice versa. Interestingly, only marginal differences were induced by low (blue) and high (red) infection-inflammation scenarios from the complement response with regard to the resulting distributions of infection scores after AM response in Game II (see Fig. 6(c,d), blue and red inline histograms). Of note, we generally observe that the level of infection is reflected by the level of inflammation (see Fig. 6(c,d)). In scenarios with low infection-dose, as shown in Fig. 6(c), AM with high activity were in the position to clear the infection in most of the cases (*p*(IS_II_ < 0.25) = 95%), in contrast to scenarios with high infection-dose (see Fig. 6(d)), where even high AM activity could only reduce but not entirely clear the infection in most of the cases (*p*(IS_II_ < 0.25) = 10%). In both infection scenarios and irrespective of AM activity, a statistically relevant number of different parameter configurations resulted in the PMN recruitment by AM to support in the elimination of the infectious agents. Interestingly, distributions of infection scores in Game II (IS_II_) generally induced qualitatively comparable distributions for the normalised inflammation (NI_II→III_).

Linking Game II and Game III by the normalised inflammation NI_II→III_, we found that, since AM with lower activity induced on average higher infection scores, they gave rise to increased levels of inflammation associated with increased PMN recruitment. In general, low infection-doses were found to be cleared for a wide range of PMN activities as indicated by infection score IS_III_ < 0.25 in Fig. 6(e). This implies, in agreement with our earlier observations (see Fig. 5(a)), that the amount of recruited PMN was always sufficient and could even compensate lower PMN activities. The situation is different for the case of high infection-doses, where a minimum-activity of PMN was required to ensure statistically firm clearance of infection (see Fig. 6(f)). Interestingly, we observed an advantageous effect for fungal clearance by PMN for AM with reduced activity in counteracting the fungal pathogens and consequently higher production of inflammatory signals increasing PMN recruitment. As can for example be seen for high PMN activity and high infection-dose, the probability for a persistent infection rose from *p*(IS_III_ ≥ 0.25) = 0.7% in cases with low AM activity to *p*(IS_III_ ≥ 0.25) = 2.5% in cases with high AM activity. This stresses once again the pivotal role of AM with regard to PMN recruitment. For low and medium PMN activity, the probability for a persistent infection *p*(IS_III_ ≥ 0.25) was ranging, respectively, from 32% to 86% and from 12% to 43%. For pathogen removal, high PMN activity was required the higher the infection-dose and the lower the AM induced inflammatory signal produced for PMN recruitment.

## Discussion

In this study, we applied EGT on graphs to investigate the dynamic counterplay between pathogens and the human immune system in various infection-inflammation scenarios. For illustrative purposes we focused on the concrete example of *A. fumigatus* infection, because even though this human-pathogenic fungus constitutes a common challenge, its potential threat can obviously be permanently managed under physiological immune conditions. Our virtual infection-inflammation model combined the three temporally distinct stages of the innate immune response against this fungal pathogen, *i.e.* the direct and immediate response by the complement system (Game I), followed by the phagocytic activity of AM (Game II) and the subsequent recruitment and immune response by PMN (Game III). Thus, going beyond separate *in silico* experiments of the three stages, we linked the three individual games in a time-ordered fashion. This is achieved by accounting for the quantitative elevation in the host inflammation, depending on the evolution of the various morphotypes that *A. fumigatus* can exhibit in the course of an infection. We performed stochastic computer simulations of various infection-inflammation scenarios scanning the parameter space of the model to identify and predict the relative importance of specific mechanisms in the innate immune response against *A. fumigatus*.

The activity of the complement system and the availability of nutrients determine the evolution of the fungal morphotypes, *i.e.* how the population of fungal cells is composed of resting and swollen conidia. In line with earlier experiments in mice, where a major inflammatory complement component (C5a) was depleted inducing a higher virulence of the pathogen^45^, it was observed in the model that conidia did shift from the resting state to the swollen state associated with a higher infection potential. It can be concluded, that in this case the pressure of the complement system became so low that the uptake of nutrients made this state transition beneficial. We also found this effect to be increased in the limit of high infection-doses.

While the complement system is believed not to contribute to direct killing of this fungus, in agreement with previous experimental investigations^29^,^30^,^46^, our simulations predicted a substantial contribution of the complement system to AM activity in AS via inflammatory signalling. Interestingly, on the one hand, it is well-known that AM comprise the largest population of resident cells in the respiratory tract^47^ and that impairment of their function is one of the risk factors for invasive mycoses^43^. On the other hand, based on experiments with mice, Mircescu *et al*. claimed that PMN but not AM play the essential role in the immune response against *A. fumigatus*^44^. Our simulations revealed that these seemingly contradictory view points could be reconciled by accounting for the impact of the infection-dose on the time course of the infection-inflammation scenario. In particular, we observed in the *in silico* experiments that AM have a key role in the regulation of the immune response and that this regulatory effect depended on the strength of the infection-dose. As a matter of fact, infections could generally be cleared by AM alone and without considerable PMN recruitment in the limit of low infection-doses corresponding to the typical dose of daily inhalation, where AM were mainly confronted with conidia in the resting and swollen state. In contrast, higher infection-doses were associated with higher inflammation and, consequently, inflammation-dependent PMN recruitment by AM was pronounced, such that PMN but not AM were ultimately playing the essential role in the phagocytosis of the fungal cells. This simulation-derived interpretation is in agreement with the fact that the study by Mircescu *et al*. was indeed performed with infection-doses that were orders of magnitude above the dosis of daily inhalation^44^.

The regulatory function of AM was of paramount importance in the simulations, due to the quantitative and qualitative limitations of AM to phagocytose and kill *A. fumigatus* in the hyphal state^39^. However, apart from AM activities directed against the pathogen itself, these immune cells do also play an important role in shutting down inflammation and in initiating tissue recovery at sites of damage in later stages^48^,^49^,^50^. PMN are essential and highly skilled phagocytes that are equipped with various antifungal capabilities that can be fungicidal and/or fungistatic^30^,^34^,^39^. Our simulation results confirmed the importance of PMN, however, their recruitment and defensive function were most important in the limit of high infection-doses. Interestingly, impairment of PMN function in the scenarios with low infection-doses was compensated by an increased recruitment of these cells, however, this could consequently imply an increase in self-damage of tissue, *e.g.* by neutrophilic respiratory burst, which constitutes a threat for the host itself 51. Nevertheless, PMN recruitment was found to play an important factor across all possible infection-doses, because lack of PMN function was associated with increased probabilities for pathogen persistence, as could be expected from findings in previous experimental studies^52^. Even though beyond the scope of the current modelling approach, it is very well conceivable that PMN recruitment by AM-induced inflammation occurs more quickly in the limit of high infection-doses.

Systems biology relies on the cross-fertilisation between theory and experiment. While uncertain or even unknown biological parameters can only be determined in wet-lab experiments, theoretical approaches can direct this exploration by generating concrete hypotheses. Obviously, parameters in the payoff matrices of EGT relate to the abstract concept of reproductive fitness in terms of nutrient uptake and immune response and, therefore, may be difficult and/or impossible to be separately measured in experiment. However, our study suggests that quantitative insights about the metabolic activities of different *A. fumigatus* morphotypes would narrow down the range of relevant nutrient contributions in the model and by that render scanning of several parameters obsolete. In turn, the model could be refined, extended and reviewed to focus investigations on particular aspects in the reduced parameter space with higher attention to details. Furthermore, guided by the outcome of our simulations, we suggest experimental investigations that may clarify the dependence between the infection-dose and the inflammatory signalling, *e.g.* by determining the cytokine profile secreted from AM and/or PMN. Similarly, *A. fumigatus* infection in mice with fully and/or partly impaired AM and/or PMN populations could as well be systematically investigated as a function of the infection-dose to specify the model dynamics of infection-inflammation scenarios. These investigations could be performed using different microscopy techniques: light-sheet microscopy could reveal cellular distributions, i.e. of conidia, AM and PMN, in the lung, whereas multi-photon microscopy could even allow monitoring dynamical aspects of cells in the lung. Furthermore, processes like phagocytosis of fungal cells by AM and PMN as well as recruitment of PMN could be quantified using fluorescence-activated cell sorting (FACS) at different time points and as a function of the infection-dose. Spatial-temporal insights from imaging experiments will support realistic mathematical modeling within the image-based systems biology approach^11^.

The current version of the virtual infection-inflammation model captures the most relevant aspects of the innate immune response against *A. fumigatus*, but could be extended by other mechanisms, even including aspects of adaptive immunity, to more comprehensively resemble the full complexity of the infection process. For example, the first cells getting into physical contact with inhaled conidia are AEC of type I and type II, where type II cells were reported to internalise conidia and to generate inflammatory signals^38^. In this study, we intentionally omitted responses by AEC due to considerable gaps in our knowledge about type I AEC^53^ that, in fact, make up the alveolar surface almost entirely^54^. Dendritic cells were also shown to contribute to fungal clearance^55^ and a dysregulation in Th1/Th2 responses lead to invasive pulmonary aspergillosis despite a non-neutropenic status of the host^56^, thus, revealing substantial evidence for the importance of adaptive immune responses in *A. fumigatus* infection. In future work, extensions of the virtual model might include responses mediated by adaptive immunity to study the relative contributions from innate and adaptive immune effectors and functions. This could be realised by a network of evolutionary games, where the immune response of the host is adapting regarding the activity against pathogenic attacks. The charming aspects of EGT lie, on the one hand, in the elegant reduction of model parameters to the ratios of parameters and, on the other hand, in the inherent extensibility that allows to realistically capture the characteristic features of complex host-pathogen interactions.

## Methods

### Modelling the dynamics of host-pathogen interactions by EGT on graphs

The game-theoretical description of the infection-inflammation scenario considered in this study is constructed as a consecutive sequence of three evolutionary games (see Fig. 1(b)). These games comprise the time course of the innate immune response and are played on graphs in order to perform *in silico* experiments of *A. fumigatus* infection using a pseudo-spatial representation of AS. Here, each fungal cell represents an infectious individual that interacts with other proximal fungal cells under the elevating inflammatory response of the host. In what follows, we present a detailed description of the interaction graph construction, the distribution of different cell types in an AS, the design of the sequentially organised games and the evolutionary dynamics algorithm.

### Environmental setting: alveolar sacs

Evolutionary dynamics take place in a typical AS. AS are organisational structures in the lower respiratory tract of the lung and are composed of 21 alveoli on average^33^,^57^. Alveoli in an AS are inter-connected via alveolar entrance rings and pores of Kohn^53^,^58^, whereas different AS are considered as fairly independent units. In order to reflect connectivity properties between alveoli in a cylindrically shaped AS, we represent them by densely packed spheres as shown in Fig. 1(a). A relationship between any two neighbouring alveoli is assumed if their spheres touch each other.

### Distribution of cells in alveolar sacs

Each alveolus represents a virtual site in which typical numbers of fungal and immune cells are placed. The number of fungal cells present in one AS emerges from a dose-dependent distribution over all AS upon inhalation or upon administration. The fungal dosis ranges from *n*_dose_ = 6.3 · 10^3^ conidia (daily inhalation for humans) to *n*_dose_ = 10^7^ conidia (administration in experiments with mice). First, the number of fungal cells per AS, *n*_fc_, is drawn from the Binomial distribution:


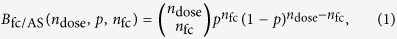


where *p* = 1/*n*_AS_ denotes the probability to choose the AS under consideration and the total number of AS is about *n*_AS_ = 2.3 · 10^7^ in humans^33^,^57^. Second, the number of fungal cells per AS, *n*_fc_, is uniformly distributed over the alveoli of the AS. Similarly, the number of resident AM (*n*_AM_) and recruited PMN (*n*_PMN_) follow the same distribution process over AS. In this case, *n*_dose_ in Eq. 1 is replaced by the total number of 2.1 · 10^9^ macrophages in humans, whereas for PMN this number can be up to eight times higher depending on the strength of PMN recruitment^40^.

### Construction of the interaction graph

The interaction graph is based on the spatial proximity between *A. fumigatus* conidia in the AS. Interactions between the fungal cells of an AS are generated in a rule-based fashion: Two fungal cells interact,

1. If they are located in the same alveolus or
2. If they are randomly selected from two neighbouring alveoli and do not yet have another inter-alveolar interaction.

These rules give rise to a proximity-based interaction graph between all fungal cells in the same alveolus or fungal cells that are nearby in neighbouring alveoli, *e.g.* reflecting intra-alveolar localisation close to one of the pores of Kohn or the alveolar entrance ring. An example for a fungal interaction graph is shown in Fig. 1(a).

### Sequential design of host-pathogen games

Fungal cells are viewed as infectious agents that play against each other for survival under the inflammatory pressure of the immune system. The different morphotypes that conidia of *A. fumigatus* can develop with time correspond to the strategies that the fungal cells may adopt in each game and are summarised in Table S1. We consider three different stages of innate immunity that are reflected by three distinct evolutionary games and that are played on the interaction graph of fungal individuals as depicted in Fig. 1(b).

In each of the three games, beneficial effects like the consumption of nutrients as well as the pressure of the immune system are aggregated in an utility function per individual, which contains quantitative information about the reproductive fitness of the fungal cell. This utility function has the general structure


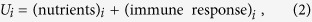


where *i* denotes the *i*-th fungal individual. Nutrients generally increase the reproductive fitness and their contributions enter the expression with a positive sign, in contrast to payoffs associated with the immune response that have values below zero. The amount of nutrient uptake by fungal individuals depends on the strategy adopted by the cell, where *E*_D_, *E*_R_, *E*_S_ and *E*_H_ denote the nutrient contributions for fungal cells in the state “dead”, “resting”, “swollen” and “hyphal”, respectively. Since the reproductive fitness of hyphae is higher than for swollen conidia and swollen conidia outperform resting conidia, we set the relation between the nutrient contributions to *E*_D_ < *E*_R_ ≤ *E*_S_ ≤ *E*_H_ with *E*_D_ = 0 for dead cells. Reduction of reproductive fitness induced by immune responses either acts on single fungal individuals or on pairs of individuals that are interacting with each other in a game-specific context.

#### Game I–Complement system

The proteins of the complement system are always present in the alveolar lining layer (surfactant) and immediately surround each fungal cell starting from its entry into the AS. Opsonisation of conidia induces inflammation and phagocyte attraction to the site of infection. This is the predominant immune response in the first minutes. At this stage, conidia of *A. fumigatus* may either stay in the “resting” state or initiate radial growth and switch to the “swollen” state. This game is restricted to these two states, because, during the first hour after entry into the lower respiratory tract, conidia do not germinate. Opsonisation and chemoattraction are represented by morphotype-specific negative payoffs as a function of the complement responses *c*_R_ and *c*_S_ that are associated with resting and swollen conidia, respectively. These effects are taken into account for each fungal individual irrespective of alveolar localisation and interaction partners. Both, resting and swollen conidia, can not evade opsonisation by the complement, but swollen conidia do activate the complement system more intensively, implying that *c*_S_ ≥ *c*_R_^36^. In addition, proximal fungal individuals interfere with each other due to superimposed inflammatory and chemoattracting complement signals and by that induce further negative payoffs *P*^I^ that depend on the number of interaction partners.

The utility function of fungal cell *i* in this game is described as


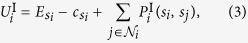


where *s*_*i*_ ∈ {R, S} is the morphotypic strategy of fungal individual *i*, 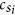 is the complement response associated with strategy *s*_*i*_, 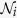 are the interaction partners of fungal cell *i* and 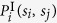 the payoffs as defined by the payoff matrix for Game I in Table 1. The utility function indicates that the more interaction partners a fungal individual has, the more it will be under pressure by complement activity.

#### Game II–Alveolar macrophages

The second game refers to the time scale of a few hours after entrance of conidia in the AS, which is the phase where germination of conidia becomes possible. In this phase, *A. fumigatus* conidia get into contact with professional phagocytes, *i.e.* AM being resident in alveoli. They are able to phagocytose conidia and have the potential to kill swollen conidia, while they fail to phagocyte fungal cells that filamented and have hyphae. The AM activity *α*_AM_ measures the ability to phagocytose and kill the fungal cells. At this stage of the immune response, conidia are enabled to adopt the four strategies “resting”, “swollen”, “hyphal” and “dead”, where the latter strategy is preferred in the case of a strong immune response that is not compensated by a nutrient contribution (*E*_D_ = 0). The number of AM present in an alveolus is drawn from a Binomial distribution, as was done previously in our agent-based virtual *A. fumigatus* infection models^16^,^17^ and as described before.

In this game, pairs of fungal cells are interacting under the pressure of the immune response mediated by AM. Each fungal cell of a pair aims to adopt a strategy that improves its reproductive fitness, at the risk of increasing the pressure by the immune system for both fungal cells. Fungal cells with no interaction partner receive the full immune exertion by AM, as if they would interact with a dead fungal cell. Thus, in the simulation algorithm a solitary fungal cell was virtually connected with a fungal cell in the state “dead”.

AM respond with morphotype-specific activities, including phagocytosis, killing, inflammation and recruitment, which are summarised in the immune responses *m*_R_, *m*_S_, *m*_H_ against resting, swollen and hyphal fungal cells, respectively. Dead fungal cells receive a zero payoff from immune activities by AM (AM response *m*_D_ = 0). The response of AM against swollen conidia *m*_S_ is set to be strongest, followed by resting conidia *m*_R_ and the hyphal morphotype *m*_H_, *i.e.*, the relation between the AM responses is given by *m*_D_ < *m*_H_ ≤ *m*_R_ ≤ *m*_S_, which is in line with experimental observations^39^.

The utility function of fungal cell *i* is defined as follows:


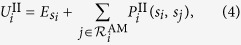


where 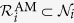 denotes the set of interactors for which the fungal pair (*i*, *j*) with 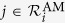 is randomly selected for AM–*A. fumigatus* interaction. The payoffs related to the AM immune response, 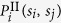, are defined in the payoff matrix shown in Table 1.

#### Game III–Polymorphonuclear neutrophils

AM have important immune regulatory functions for the recruitment of cells from the bloodstream. For example, once recruited to the site of infection, PMN have an armory of weapons to attack pathogens. They can kill the fungus intracellularly after successful phagocytosis or extracellularly by the secretion of reactive oxygen species. Furthermore, they can undergo NETosis by committing an altruistic suicide in which they release their DNA and by that trap the fungus to prevent it from further spreading. After the recruitment of PMN to lung alveoli, these phagocytes govern the immune response and their overall activity is measured by the parameter *α*_PMN_.

We introduce the parameter *ρ*_PMN_ to control the relative number of recruited PMN to the site of infection, where we impose – in agreement with observations in wet-lab experiments^40^ – an upper PMN recruitment limit of eight times the number of present AM. As in Game II, pairs of conidia interact with PMN in a randomised fashion and can adopt the four strategies “resting”, “swollen”, “hyphal” and “dead” in this game. However, in contrast to Game II, the various defence mechanisms of PMN give rise to different relations in their specific responses. The relations between the PMN responses *g*_R_, *g*_S_ and *g*_H_ against fungal cells in the resting, swollen and hyphal state are given by *g*_R_ ≤ *g*_S_ ≤ *g*_H_^29^. The utility function of fungal cell *i* in Game III is given by


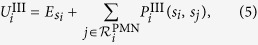


where 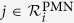 defines the interaction pairs (*i*, *j*), which randomly interact with a PMN. 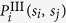 is the payoff imposed by interaction with a PMN as defined in Table 1.

The three games are linked in sequential order – Game I → Game II → Game III – to investigate the counterplay of *A. fumigatus* infection and host inflammation. Game linking is associated with the inflammatory signalling in response to the infectious agent, initiating the next higher instance along the cascade of the innate immune response. To this end, we introduce measures that enable quantifying the degree of infection and inflammation.

### Quantification of infection and inflammation for game linking

To quantify the degree of infection induced by the human-pathogenic fungus *A. fumigatus* we define the infection score (IS) that is computed after execution of game *G*:


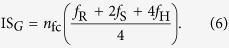


Here *n*_fc_ denotes the number of fungal cells in the AS and *f*_R_, *f*_S_, *f*_H_ and *f*_D_ with *f*_R_ + *f*_S_ + *f*_H_ + *f*_D_ = 1 refer to the fractions of fungal cells in the resting, swollen, hyphal and dead state, respectively. By construction, the infection score assumes increasing values IS_*G*_ = *n*_fc_/4, IS_*G*_ = *n*_fc_/2 and IS_*G*_ = *n*_fc_ for populations consisting of increasingly challenging morphotypes *f*_R_ = 1, *f*_S_ = 1 and *f*_H_ = 1, respectively. Furthermore, particular infection scores IS_*G*_ correspond to specific numbers of persisting fungal cells, irrespective of the infection-dose. For example, IS_*G*_ = 0.5 either corresponds to one fungal cell in the swollen state or to two conidia in the resting state, whereas IS_*G*_ = 0.25 represents one remaining fungal cell that is in the resting state. This threshold, IS_*G*_ = 0.25, is set in the further analysis to distinguish between persistent (IS_*G*_ ≥ 0.25) and cleared (IS_*G*_ < 0.25) infections.

Game linking is associated with the inflammatory signalling in response to the infectious agent. In particular, we link the complement system (Game I) with AM response (Game II) by the normalised inflammation (NI) measure that is defined as


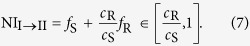


Based on literature data^36^, this measure accounts for the relative difference in the complement response against the fractions of resting (*f*_R_) and swollen (*f*_S_) conidia. Furthermore, NI_I→II_ accounts for relative differences in the complement response against resting and swollen conidia by the scaling factor *c*_R_/*c*_S_. The normalised infection value NI_I→II_ regulates the response of AM in Game II by scaling the AM activity *α*_AM_: 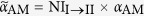. Similarly, we link Game II to Game III by modelling PMN recruitment to depend on AM-secretion of the PMN-chemoattractant MIP-1. The secretion of MIP-1 is known to depend on the different *A. fumigatus* morphotypes^40^. Thus, we consider the relative recruitment number *ρ*_PMN_ = NI_II→III_, with the normalised inflammation:


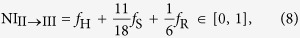


where the coefficients conserve the relative responses of AM to MIP-1 for the different *A. fumigatus* morphotypes, as deduced from Ref. 40. Note that, in case the *A. fumigatus* infection can be cleared by AM, *i.e.* all fungal cells are dead *f*_D_ = 1 and *f*_R_ = *f*_S_ = *f*_H_ = 0 after Game II was played, no PMN-chemoattractant would be secreted and no PMN would be recruited.

### Evolutionary dynamics

In each game, iterations over repetitive evolutionary steps *t*_evo_ are performed as depicted in Fig. S1. To ensure equilibration of the evolutionary dynamics, we set the number of iterations to 10^4^. In each iteration step fungal cells receive nutrients and are confronted with the respective immune response. Each individual fungal cell evaluates its utility function in every time step *t*_evo_ containing the information on its reproductive fitness. Mutation and adaptation are evolutionary mechanisms performed in response to differences in the utility functions of individuals in the following way.

#### Mutation

Random change between strategies with probability *p* = 0.01.

#### Adaptation

Probabilistic change of an individual’s current strategy *s*_*i*_ into a strategy *s*′ applied by at least one fungal individual in the neighbourhood with higher reproductive fitness. Proportional imitation is applied as microscopic update rule and a distinction is drawn between the strategy “dead” (*s*_dead_) and strategies related to individuals that are alive:

**Case** *s*′ ≠ *s*_dead_: if strategy *s*′ is played in the neighbourhood 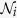 of individual *i*, it adopts strategy *s*′ with probability:


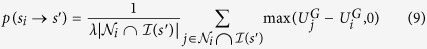


where 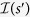 is the set of all individuals playing strategy *s*′ and *λ* a normalisation factor to ensure *p*(*s*_*i*_ → *s*′) ∈ [0, 1].

**Case** *s*′ = *s*_dead_:


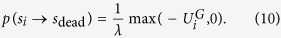


### Statistical measures

In order to distinguish parameters of the games by their relevance for the outcome of the infection-inflammation scenarios, we compute the mutual information (MI) as follows:


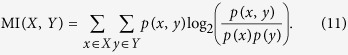


Here, *X* is the set of values related to one unknown parameter and *Y* the corresponding set of infection scores IS_*G*_ of game *G*. Furthermore, *p*(*x*, *y*) denotes the joint probability function, *p*(*x*) and *p*(*y*) are marginal probability distributions of *X* and *Y*, respectively^59^. Since IS_*G*_ is a continuous measure, *Y* is discretised using different bin sizes for the calculation of MI(*X*, *Y*). We checked that the dependence of the mutual information MI(*X*, *Y*) on the binning of the infection score IS_*G*_ does not affect the ranking of the most important parameters. These are the AM activity *α*_AM_ in Game II (see Table S2 and Fig. S2 (a)) and both PMN activity *α*_PMN_ and PMN recruitment *ρ*_PMN_ in Game III (see Table S2 and Fig. S2 (b)).

### Implementation and Simulation

All parameters used in the evolutionary games are scanned in reasonable ranges to capture their influence on the outcome of each game and the ultimate outcome of the infection. The evolutionary game-theoretical algorithm is implemented in the object-oriented programming language C++ and simulations are performed by the simulation algorithm depicted in Fig. S1. Statistical analyses are carried out using R. To reduce effective runtime, the algorithm is parallelised and is executed on a SUSE Linux Enterprise Server version 11 based on a x86-64 architecture with 512 GB RAM and 48 AMD Opteron processors, each running on 1781 MHz.

Virtual infection-inflammation scenarios were performed for different fungal doses and varied parameter configurations. To account for the stochastic nature of the *in silico* experiments, 100 repetitions were executed and from each simulation long-term density distributions of resting and swollen conidial fractions with their related averages and standard deviations were extracted.

## Additional Information

**How to cite this article**: Pollmächer, J. *et al*. Deciphering the Counterplay of *Aspergillus fumigatus* Infection and Host Inflammation by Evolutionary Games on Graphs. *Sci. Rep.* **6**, 27807; doi: 10.1038/srep27807 (2016).

## Supplementary Material

Supplementary Information

## Figures and Tables

**Table 1:** Payoff matrices for Game I, II and III.

		*s*_2_			
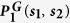		R	S	H	D
Game I					
*s*_1_	R	−*c*_R_/2	−*c*_S_/2	–	–
	S	−*c*_R_/2	−*c*_S_/2	–	–
	H	–	–	–	–
	D	–	–	–	–
Game II					
*s*_1_	R	−1/2 × *m*_R_	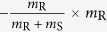	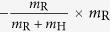	−*m*_R_
	S	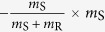	−1/2 × *m*_S_	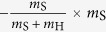	−*m*_S_
	H	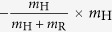	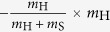	−1/2 × *m*_H_	−*m*_H_
	D	0	0	0	0
Game III					
*s*_1_	R	−1/2 × *g*_R_	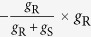	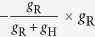	−*g*_R_
	S	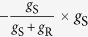	−1/2 × *g*_S_	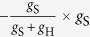	−*g*_S_
	H	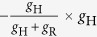	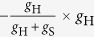	−1/2 × *g*_H_	−*g*_H_
	D	0	0	0	0

Payoffs 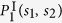, 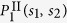 and 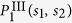 of Game I, Game II and Game III are shown for fungus 1 interacting with fungus 2. R, S, H and D denote resting, swollen, hyphal and dead fungal cells, respectively. The complement response for resting and swollen fungal cells in the Game I are represented by *c*_*R*_ and *c*_*S*_, respectively. *m*_H_, *m*_R_, *m*_S_ and *g*_R_, *g*_S_, *g*_H_ are the responses by AM or PMN on encounter of resting condia, swollen conidia or hyphae. The complement responses satisfy the condition *c*_R_ ≤ *c*_S_ as well as the AM responses *m*_H_ ≤ *m*_R_ ≤ *m*_S_ and the PMN responses *g*_R_ ≤ *g*_S_ ≤ *g*_H_, which determine the payoffs.
