# Supplementary material for: Deciphering the Counterplay of Aspergillus fumigatus Infection and Host Inflammation by Evolutionary Games on Graphs
Source: Sci Rep. 2016 Jun 13;6:27807. doi: 10.1038/srep27807 (PMC4904243; doi:10.1038/srep27807)
Supplement: Supplementary Information [file srep27807-s1.pdf]

Supplementary Information

Deciphering the Counterplay of *Aspergillus fumigatus* Infection and Host Inflammation by Evolutionary Games on Graphs

Johannes Pollmächer[1,2], Sandra Timme[1,2], Stefan Schuster[3], Axel A. Brakhage[2,4], Peter F. Zipfel[2,5], and Marc Thilo Figge[1,2,*]

[1]Research Group Applied Systems Biology, Leibniz Institute for Natural Product Research and Infection Biology – Hans Knöll Institute, Jena, Germany
[2]Faculty of Biology and Pharmacy, Friedrich Schiller University Jena, Germany
[3]Department of Bioinformatics, Faculty of Biology and Pharmacy, Friedrich Schiller University Jena, Germany
[4]Department of Molecular and Applied Microbiology, Leibniz Institute for Natural Product Research and Infection Biology – Hans Knöll Institute, Jena, Germany
[5]Department of Infection Biology, Leibniz Institute for Natural Product Research and Infection Biology – Hans Knöll Institute, Jena, Germany
[*]E-mail: thilo.figge@leibniz-hki.de

Table S1: Game-dependent morphotype options of *A. fumigatus*.

	A. fumigatus strategy/morphotype			
permitted morphotype (yes/no)	resting (R)	swollen (S)	hyphal (H)	dead (D)
Game I: complement system	yes	yes	no	no
Game II: AM	yes	yes	yes	yes
Game III: PMN	yes	yes	yes	yes

Possible strategies for fungal cells in the three different games. In Game I only resting and swollen conidia occur, while in Game II and Game III all four strategies are possible.

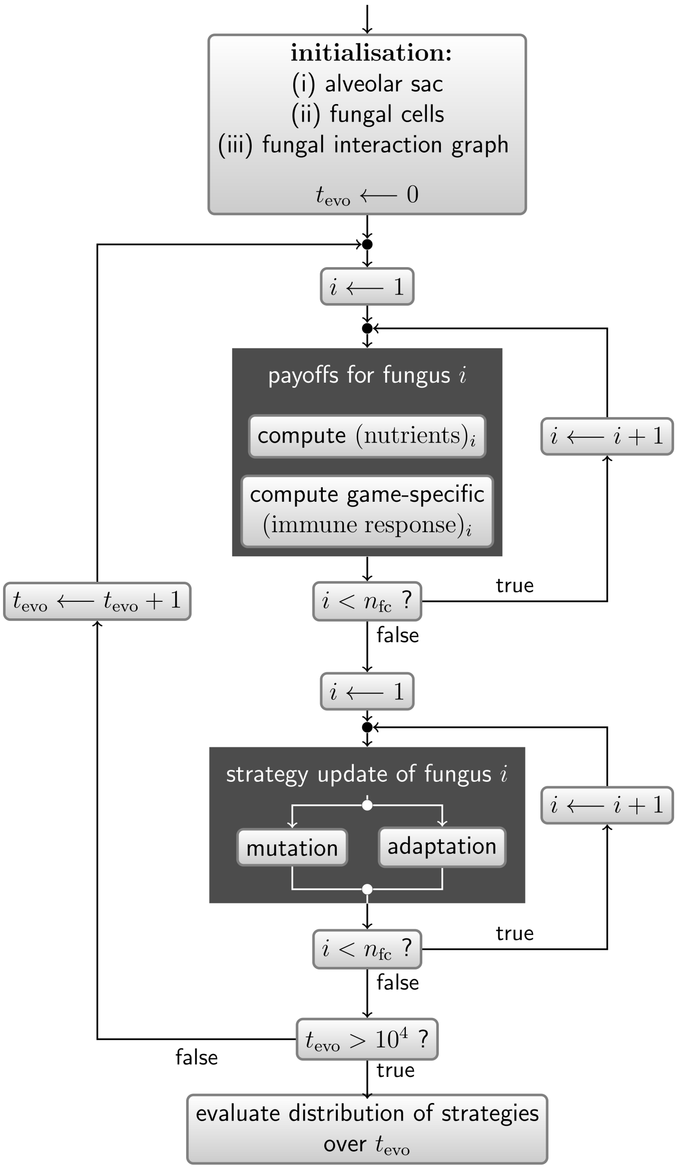

Figure S1: Simulation algorithm of each evolutionary game. Schematic overview of the simulation algorithm as applied for each evolutionary games on the fungal interaction graphs in alveolar sacs. Iterations over evolutionary steps t_{evo} include the computation of payoffs per fungus and microscopic strategy updates based on the concepts of mutation and adaptation. The number of fungal cells for which simulations are performed in the alveolar sac is denoted by n_{fc}.

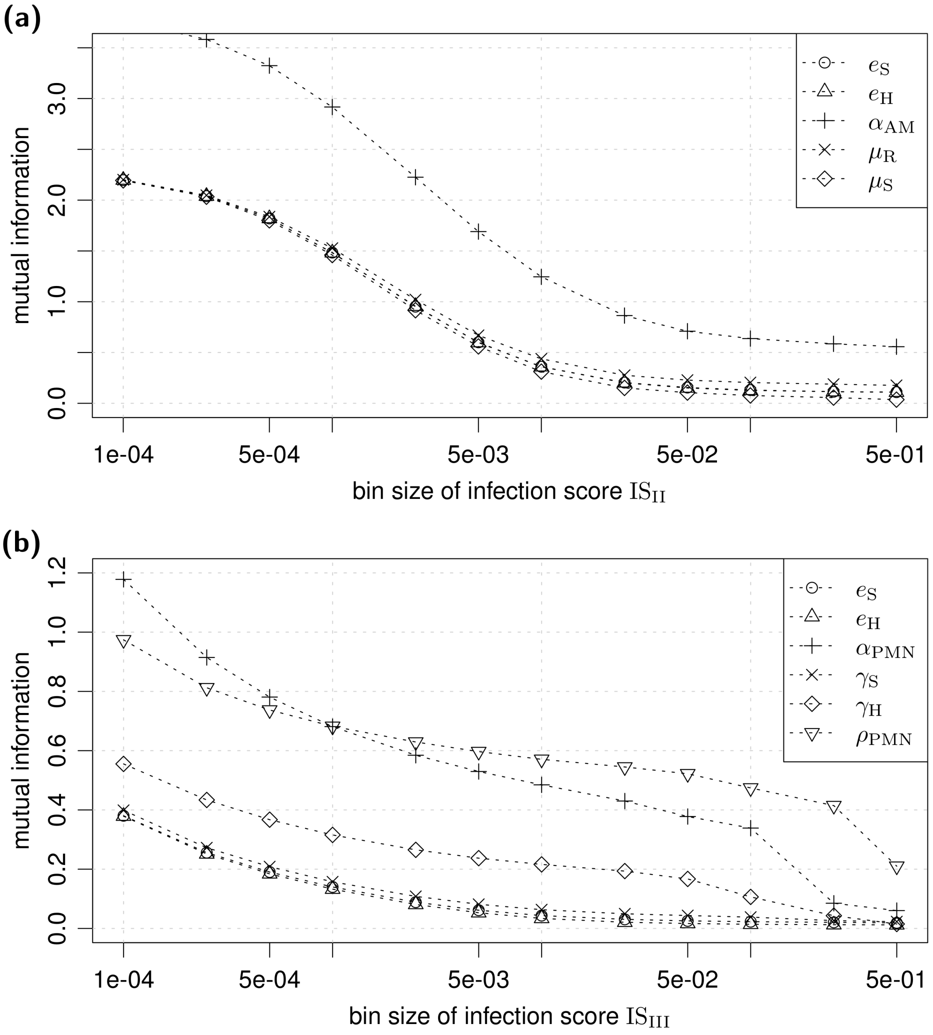

Figure S2: Dependence of mutual information on the bin size. Mutual information for the parameters of (a) Game II and (b) Game III as a function of the bin size of the infection score.

Table S2: Mutual information between infection score and parameters of Game II and Game III.

parameter	condition	MI (Game II)	MI (Game III)
$e_\mathrm{S} = E_\mathrm{S}/E_\mathrm{R}$	$e_\mathrm{S} \geq 1$	0.3679	0.0434
$e_\mathrm{H} = E_\mathrm{H}/E_\mathrm{S}$	$e_\mathrm{H} \geq 1$	0.3463	0.0338
$\alpha_\mathrm{AM} = m_\mathrm{R}/E_\mathrm{R}$		**1.1439**	–
$\mu_\mathrm{R} = m_\mathrm{R}/m_\mathrm{H}$	$\mu_\mathrm{R} \geq 1$	0.3220	–
$\mu_\mathrm{S} = m_\mathrm{S}/m_\mathrm{R}$	$\mu_\mathrm{S} \geq 1$	0.3324	–
$\alpha_\mathrm{PMN} = g_\mathrm{R}/E_\mathrm{R}$		–	**0.4847**
ρ_PMN	$\rho_\mathrm{PMN} \in [0, 1]$	–	**0.5708**
$\gamma_\mathrm{S} = g_\mathrm{S}/g_\mathrm{R}$	$\gamma_\mathrm{S} \geq 1$	–	0.0632
$\gamma_\mathrm{H} = g_\mathrm{H}/g_\mathrm{S}$	$\gamma_\mathrm{H} \geq 1$	–	0.2161

The mutual information (MI) as a function of the infection score (IS) was computed for bin size 0.01 and for each of the games parameters. E_R, E_S, E_H describe the nutrient contributions for resting, swollen and hyphal fungal cells, respectively. μ_R, μ_S and γ_R, γ_S are fractions of response variables for the encounter of resting and swollen conidia and hyphae by either AM or PMN. *Bold* numbers denote the most relevant parameters of the respective evolutionary game, which are the AM activity α_AM in Game II and both PMN activity α_PMN and PMN recruitment ρ_PMN in Game III.
